# Supplementary material for: Single-cell analysis of patient-derived PDAC organoids reveals cell state heterogeneity and a conserved developmental hierarchy
Source: Nat Commun. 2021 Oct 5;12:5826. doi: 10.1038/s41467-021-26059-4 (PMC8492851; doi:10.1038/s41467-021-26059-4)
Supplement: Supplementary file 1 — Supplementary Information [file 41467_2021_26059_MOESM1_ESM.pdf]

Supplementary Material

Single-cell analysis of patient-derived PDAC organoids reveals cell state heterogeneity and a conserved developmental hierarchy

Teresa G Krieger[1,2,†], Solange Le Blanc[3,4,5,†], Julia Jabs[2,†], Foo Wei Ten[1,2], Naveed Ishaque[1], Katharina Jechow[1,2], Olivia Debnath[1], Carl-Stephan Leonhardt[3], Anamika Giri[2], Roland Eils[1,2,*], Oliver Strobel[3,5,6,*], Christian Conrad[1,2,*]

[1]Digital Health Center, Berlin Institute of Health (BIH)/Charité-Universitätsmedizin Berlin, Berlin, Germany
[2]Division of Theoretical Bioinformatics, German Cancer Research Center (DKFZ), Heidelberg, Germany
[3]European Pancreas Center, Department of General Surgery, Heidelberg University Hospital, Heidelberg, Germany
[4]Division of Molecular Oncology of Gastrointestinal Tumors, German Cancer Research Center (DKFZ), Heidelberg, Germany
[5]National Center for Tumor diseases (NCT), Heidelberg, Germany
[6]Division of Visceral Surgery, Department of General Surgery, Medical University of Vienna, Vienna, Austria

† These authors contributed equally.
* These authors jointly supervised this work.

Supplementary Figure 1: Single-cell RNA sequencing of PDAC organoids

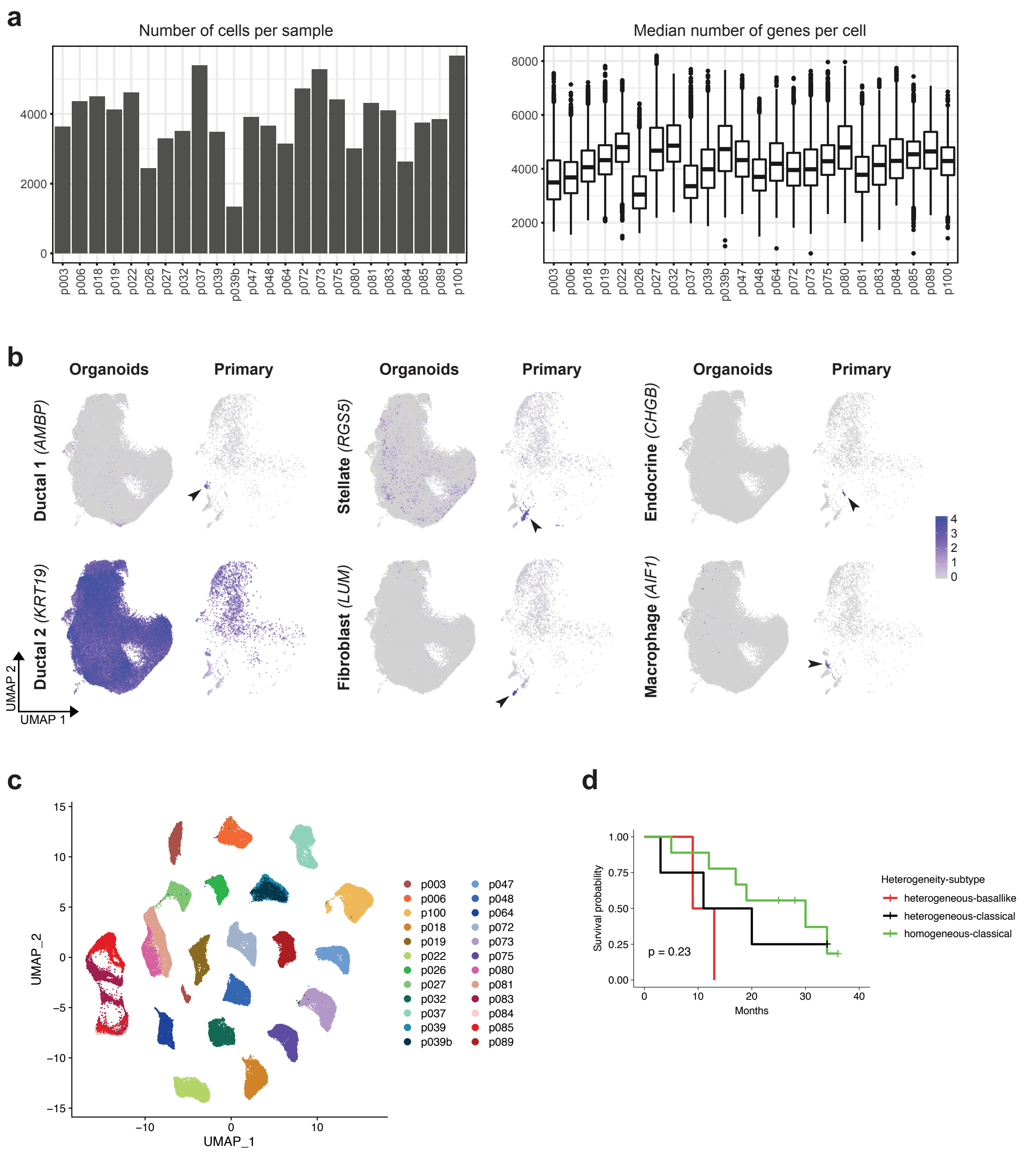

a) The bar plot indicates the number of cells (n) per sample across the PDAC organoid scRNA-seq dataset. The box plot indicates the median number of genes per cell (centre: median, lower and upper hinges: first and third quartiles, whiskers: largest and smallest values no further than 150% of the inter-quartile range from the hinges, points: data beyond whiskers plotted individually as outliers).

b) Single-cell expression of cell type marker genes in PDAC organoid cells (left) and cells from primary PDAC biopsies (right). Arrowheads indicate gene expression in small subsets of cells in primary PDAC samples, but not in organoids.

c) UMAP embedding of 24 scRNA-seq samples from 18 patients, after removal of known expression quantitative trait loci from histologically normal pancreatic tissue samples or PDAC tumor-derived tissue samples [1] from the analysis. As also shown in Figure 1c, cells cluster by patient origin (p039 is a technical replicate of p039b, p080 is a biological replicate of p081, p084 and p085 are liver metastases matched to p083).
d) Kaplan-Meier curve for our cohort, split by PDAC subtype and heterogeneity. Homogeneous classical tumor patients tended to have a better outcome whereas the outcome of basal-like tumors tended to be poorest. While this result is not statistically significant ($p=0.23$, log-rank test), reflecting that the size of our cohort is insufficient for population-based analyses, it is consistent with previous reports from larger cohorts [2,3,4,5].

Supplementary Figure 2: Distinct cell states in PDAC

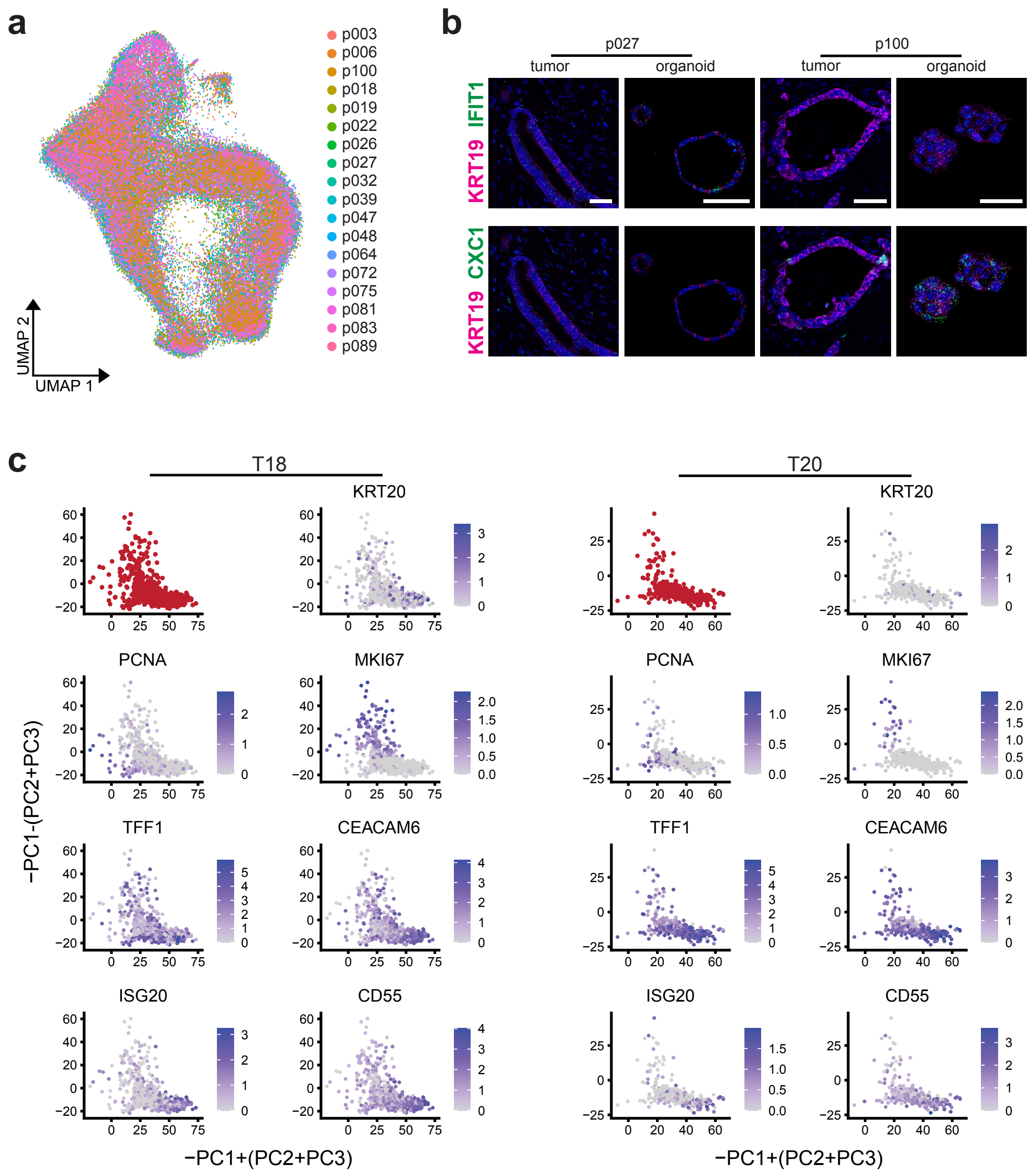

a) UMAP representation of PDAC transcriptomes from 18 primary PDAC organoid lines, after PCA-based integration. Colors indicate sample identity.
b) Representative images of RNA-FISH staining for KRT19 (magenta) together with either IFIT1 or CXC1 (green) in sections from organoids and primary tumours from p027 and p100. Nuclei are stained with DAPI (blue). Scale bars, 50 µm. FISH staining was repeated twice on individual sections with similar results.
c) Projection of scRNA-seq data from primary PDAC samples T18 and T20, published previously [6], onto the same linear combination of principal components as presented in Figure 4f (red). Expression of genes characteristic for cycling and differentiating cells is also shown.

Supplementary Figure 3: Cycling and differentiating cells in PDAC organoids and metastases

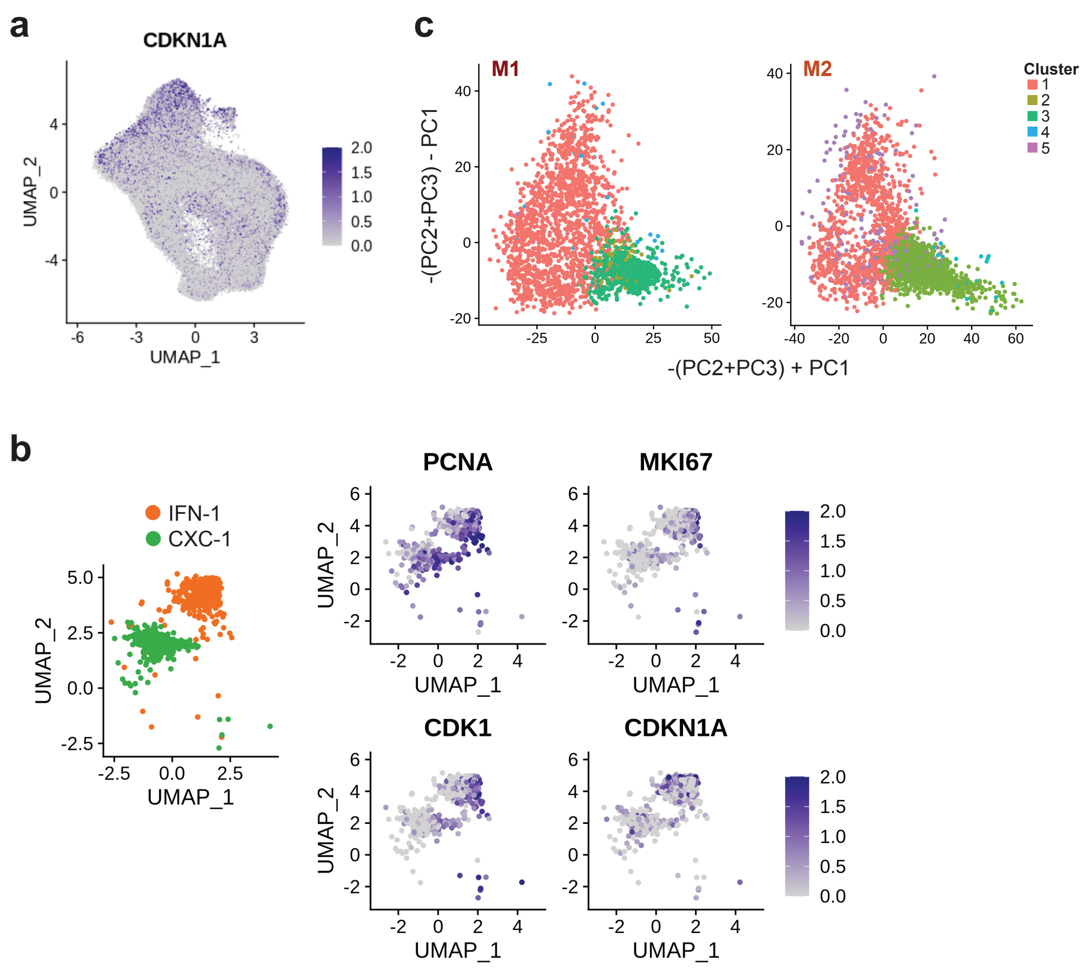

a) Expression of CDKN1A (coding for p21) across all primary PDAC organoids. The UMAP representation shown here is the same as in Figure 3c.
b) For clarity, clusters IFN-1 and CXC-1 are depicted without the other clusters, using the same UMAP representation as above (left). Expression of cell cycle related genes across these clusters indicates that they contain both cycling and quiescent cells (right).
c) Projection of M1 and M2 transcriptomes onto the primary PDAC organoid data (see Figure 4f and Methods) shows distribution of cells across the functional clusters identified in metastases (see Figure 5a).

Supplementary Figure 4: Drug responses of PDAC organoids

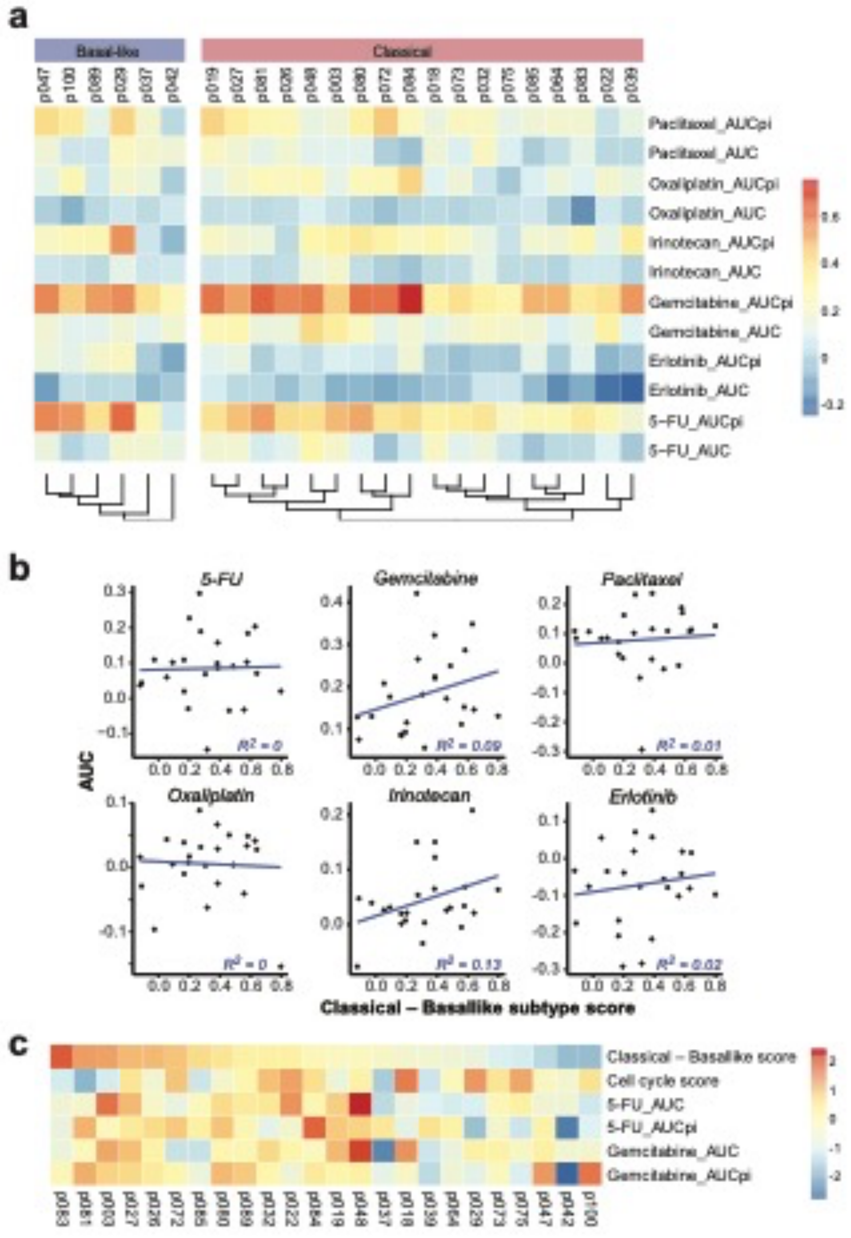

a) Drug response as measured by the area under curve for cell death (AUC) and proliferation inhibition (AUCpi) after 72 hours of treatment, for six different drugs in clinical use. Trees represent hierarchical clustering results for 'classical' and 'basal-like' PDAC organoid lines separately.
b) Correlation between median subtype scores calculated from scRNA-seq data and drug-induced cell death, as measured by the area under curve (AUC), for 24 PDAC organoid lines. A higher score on the x axis represents a more 'classical' transcriptional phenotype.
c) Overview of variation in transcriptomic PDAC subtype, median cell cycle score, as well as cell death (AUC) and proliferation inhibition (AUCpi) as measured by the area under curve in response to 5-FU or gemcitabine treatment, shown as z-scores scaled across all samples.

Supplementary Table 1: Sample information

								Bulk RNA-seq				Bulk RNA-seq based subytype assignment for original tumors
ID	Sample	Location	Morphology	Grading	IPMN context	Chemo-therapy	KRAS mutation	Classical score	Basal-like score	Moffitt subtype (2-class)	Moffitt subtype (3-class)	
pdac03	p003	Tail	hollow	G3	NO	NO	Q61H	0.0089515	0.0043576	Classical	Classical	NA
pdac06	p006	Head	hollow+filled	G3	YES	NO	G12D	NA	NA	NA	NA	NA
pdac18	p018	Tail	multLum	G3	NO	NO	G12V	0.0088198	0.0026988	Classical	Classical	NA
pdac19	p019	Tail	hollow	G2	NO	NO	G12V	0.009439	0.0024683	Classical	Classical	NA
pdac22	p022	HeadBody	mixed	G4	NO	NO	G12R	0.0090398	0.002465	Classical	Classical	Classical
pdac26	p026	Head	multLum	G2	NO	NO	G12D	0.0093641	0.003476	Classical	Classical	NA
pdac27	p027	Head	hollow	G2	NO	NO	G12V	0.0086201	0.0038162	Classical	Classical	Classical
pdac32	p032	Tail	hollow	G3	NO	NO	G12D	0.0079825	0.0040549	Classical	Classical	Classical
pdac37	p037	PeripancreaticMet	mixed	NA	YES	YES	G12D	0.0049682	0.0064576	Basal-like	Basal-like	Basal-like
pdac39	p039	Head	mixed	G2	NO	NO	G12R	0.0076139	0.0040019	Classical	Classical	NA
pdac47	p047	Head	hollow	G3	NO	NO	G12V	0.0057914	0.0069626	Basal-like	Intermediate	Basal-like
pdac48	p048	Head	mixed	G3	NO	NO	G12V	0.0086943	0.0041905	Classical	Classical	NA
pdac64	p064	Head	multLum	NA	NO	YES	G12V	0.0069608	0.0037311	Classical	Classical	NA
pdac72	p072	HeadBodyTail	mixed	NA	NO	YES	G12R	0.0084005	0.0039216	Classical	Classical	NA
pdac73	p073	PeritonealMet	multLum	NA	YES	NO	G12V	0.0078166	0.0040776	Classical	Classical	NA
pdac75	p075	Body	mixed	NA	NO	YES	G12R	0.0063818	0.004835	Classical	Classical	NA
pdac89	p089	Head	hollow	G3	NO	NO	G12D	0.0052747	0.0076665	Basal-like	Intermediate	NA
pdac80	p080	Head	hollow	G2	NO	NO	G12R	0.0090161	0.0032927	Classical	Classical	NA
pdac81	p081	Head	hollow	G2	NO	NO	G12R	0.0108247	0.0011815	Classical	Classical	Classical
pdac83	p083	Tail	mixed	G2	NO	NO	G12D	0.0112192	0.0013463	Classical	Classical	Classical
pdac84	p084	LiverMet	mixed	NA	NO	NO	G12D	0.0086887	0.0024852	Classical	Classical	Classical
pdac85	p085	LiverMet	multLum	NA	NO	NO	G12D	0.0087596	0.0039757	Classical	Classical	NA
pdac100	p100	Head	filled	G3	NO	NO	G12V	0.0032967	0.0097682	Basal-like	Basal-like	Basal-like

pdac81, pdac83 and pdac84 were derived from the same patient.
pdac80 and pdac81 were derived from the same patient.
Samples were derived from 8 male and 12 female patients.

Supplementary Table 2: Patient-specific genes

Top 20 patient-specific genes (with log[fold change]>0.25 and adjusted p-value<0.001), as shown in Figure 1d.

#	p003	p006	p018	p019	p022	p026	p027	p032	p039	p047	p048	p064	p072	p075	p081	p083	p089	p100
1	MUC5B	LCN2	DDIT4	CA9	LDHB	HIST1H1C	MMP1	CLDN3	OLFM4	CST6	ORM1	AKR1C4	KRT13	PON2	FABP1	RP11-1143G9.4	LY6D	S100A9
2	TFF2	LYZ	TMEM54	TPM2	SERPINA1	HIST1H2BK	LCN2	BMP4	RNASE1	ALPPL2	RPS4Y1	MAGEA4	RPS4Y1	MCM7	PRSS1	TFF1	CD70	S100A2
3	TFF3	SNCG	ANXA13	PLA2G2A	LINC00665	TM4SF4	PIGR	FAM3D	HLA-B	MMP7	LTB	MAGEA3	HSBP1L1	TRIP6	REG4	RGS5	CEACAM5	SPP1
4	CDKN2A	ISG15	ID1	PRSS1	HMGCS2	LINC00152	TFF2	IDH2	WFDC2	RBP1	PHGR1	AKR1C3	TESC	POP7	TM4SF20	GSTM4	PSCA	SCGB1A1
5	SCD	TFF3	CALB2	JUN	REG4	SPINK1	DMBT1	PPAP2C	IFITM3	IFI27	FABP1	AKR1C2	REG4	ACN9	TFF1	LYZ	KRT20	NTS
6	TFF1	ALDH1A1	EIF4EBP1	EDN1	CAPNS1	JUND	MTRNR2L8	SLC12A2	CXCL17	IFI6	DLD	MAGEA6	CTSE	GNB2	CBS	PGC	KLK6	KRT23
7	PSCA	ZG16B	PSAT1	CTTN	CLDN2	MT1X	LYPD2	MTRNR2L1	PLAT	DKK1	GSTM3	CES1	AQP5	TSC22D4	CD55	TFF2	JUNB	KRT15
8	CLIC3	LYPD2	TMEM45B	MT1E	GAL	HIST1H2BJ	LYZ	CEACAM5	MMP7	ISG15	CDKN2A	AKR1B10	TXNL4A	TAF6	MUC13	ANPEP	GABRP	FGFBP1
9	ADIRF	RPS4Y1	PYCR1	MYEOV	CLDN18	AC006262.5	MUC1	SRD5A3	HSPA1A	MT2A	CTNNBIP1	CHCHD10	FBP1	PILRB	PRSS3	CA2	ARHGDIB	CSTA
10	CAPS	CDKN2A	NT5C	SPINK4	RRP7A	MUC5AC	CD74	PPP1R1B	NGFRAP1	HPGD	CXCL3	PI3	SYT8	BAIAP2L1	PI3	KLK1	LDHB	S100A8
11	XIST	MUC5B	CLDN3	ACTL8	SYNE4	SMIM22	LGALS2	STXBP6	RARRES3	LYPD2	EMG1	C12orf75	TMEM176B	IFITM2	MRPS31	AKR1B10	NR4A1	CALB1
12	LGALS1	AKR1C3	GSTM3	OLFM4	MTHFS	C8orf59	HLA-DRA	GMDS	BPIFB1	KLK6	ARL14	MIB2	ADIRF	SLC25A13	PHGR1	TFF3	TCN1	ALDH3A1
13	UPK3B	CAPS	ID3	FADD	PSENEN	MIR4435-1HG	VSIG2	GCNT3	HIF1A	ANXA3	EEF1E1	CSAG1	TFF2	CPSF4	ELF1	REG4	PLAT	KRT5
14	AQP5	PI3	KRTCAP3	ERRFI1	UPK1B	ARL14	MUC13	KLK6	PRSS21	F3	IL1R2	RARRES1	PEPD	ANXA1	CEACAM5	GMDS	CEACAM6	AQP3
15	ASRGL1	AQP5	IDH2	BTG1	RBM42	KLF2	CDC42EP1	PLIN2	DMBT1	LY6D	BIRC3	MAGEA10	CLTB	ATF3	TRIM31	SELM	CDKN1A	KRT6A
16	IGFBP7	MUC1	SLC22A18	MCL1	SHKBP1	CLDN18	HLA-DRB1	KRT6B	CD74	HLA-B	MRPS18C	G6PD	IMPA2	DBF4	WBP4	SULT1C2	PLBD1	GLUL
17	BTBD16	CRIP2	SLC3A2	KLF6	KDELR3	RHOB	TCEA3	MRPL15	IFITM2	BST2	CALB2	CLDN3	TUBA4A	MOSPD3	MIPEP	AKR7A3	GCNT3	MIR205HG
18	TM4SF5	RSPH1	PLEK2	HIST1H1C	CDKN2A	RP11-462G2.1	FOS	LCN2	SERPINA1	KLK10	TPD52	TM4SF20	KRT20	CYR61	IFITM2	IL1R2	LGMN	DHRS9
19	FBXO2	SLC12A2	GSTO2	RHOB	QPRT	MT1E	TMC5	TPM2	IFI27L2	RARRES3	SMIM20	PGD	PXMP2	KRT20	ABHD3	CES2	PPP1R15A	SLPI
20	DPYSL2	RARRES3	RP11-345J4.5	RBP4	CYB5R3	SFTA2	CRIP2	PHLDA1	GSTA1	CAV1	PRSS21	CES2	TMEM176A	CTGF	IL32	OTC	LIPC	ADIRF

Supplementary Table 3: Moffitt subtype and heterogeneity scores

Moffitt subtype and heterogeneity scores based on scRNA-seq
for PDAC organoids derived from primary tumors

ID	Sample	Basal-like score	Classical score	Heterogeneity class	Moffitt subtype	Grading
pdac03	p003	0.008241758	0.991758242	homogeneous	classical	G3
pdac06	p006	0.009630819	0.990369181	homogeneous	classical	G3
pdac18	p018	0.088928413	0.911071587	homogeneous	classical	G3
pdac19	p019	0.070736434	0.929263566	homogeneous	classical	G2
pdac22	p022	0.063488624	0.936511376	heterogeneous	classical	G4
pdac26	p026	0.061140747	0.938859253	homogeneous	classical	G2
pdac27	p027	0.005461165	0.994538835	homogeneous	classical	G2
pdac32	p032	0.033864542	0.966135458	heterogeneous	classical	G3
pdac39	p039	0.16824305	0.83175695	homogeneous	classical	G2
pdac47	p047	0.615404299	0.384595701	heterogeneous	basallike	G3
pdac48	p048	0.106732348	0.893267652	homogeneous	classical	G3
pdac64	p064	0.166189112	0.833810888	homogeneous	classical	NA
pdac72	p072	0.00995341	0.99004659	homogeneous	classical	NA
pdac75	p075	0.40199637	0.59800363	heterogeneous	classical	NA
pdac81	p081	0.002322341	0.997677659	homogeneous	classical	G2
pdac83	p083	0.016581322	0.983418678	homogeneous	classical	G2
pdac89	p089	0.032509753	0.967490247	heterogeneous	classical	G3
pdac100	p100	0.810901394	0.189098606	heterogeneous	basallike	G3

Supplementary Table 4: Cluster-specific genes

Top 20 differentially expressed genes for all clusters
(with log[fold change]>0.25 and adjusted p-value<0.001)

	**Clusters**								
#	**Cycling-1**	**Cycling-2**	**Cycling-3**	**Cycling-4**	**Cycling-5**	**Noncycling-1**	**Noncycling-2**	**IFN-1**	**CXC-1**
1	PCNA	HIST1H4C	UBE2C	CCNB1	PTTG1	LYZ	PHGR1	ISG15	CXCL1
2	GINS2	RRM2	KPNA2	CDC20	CDC20	ALDH1A1	PSCA	OASL	CXCL3
3	MCM7	CDK1	CDK1	CKS2	HMGN2	SLC12A2	TFF1	IFIT3	CXCL2
4	MCM3	KIAA0101	CCNB1	PTTG1	CDKN3	TFF3	C15orf48	IFIT2	NFKBIA
5	FEN1	HMGB2	UBE2S	CCNB2	CCNB1	RNASE1	SDCBP2	ISG20	IL8
6	MCM5	ZWINT	CKS2	AURKA	BIRC5	MUC5B	TM4SF4	RARRES3	CCL20
7	SLBP	UBE2T	PLK1	UBE2S	CCNB2	AQP5	IL32	IFIT1	BIRC3
8	MCM6	MCM7	CDC20	CDKN3	STMN1	CAPS	CEACAM5	ZC3HAV1	ZC3H12A
9	DUT	TK1	TOP2A	CENPF	ILF2	OLFM4	CEACAM6	PMAIP1	TNFAIP2
10	UNG	DUT	HMGB2	PLK1	HNRNPH3	TFF2	ISG20	HERC5	IER3

Supplementary Table 5: Number of cells represented in each cluster for each patient

ID	Sample	Cluster								
		Cycling-1	Cycling-2	Cycling-3	Cycling-4	Cycling-5	Noncycling-1	Noncycling-2	IFN-1	CXC-1
pdac03	p003	549	234	188	89	426	1057	919	5	26
pdac06	p006	486	220	270	108	690	1425	753	217	35
pdac18	p018	798	889	822	201	611	396	485	65	31
pdac19	p019	696	365	392	137	551	1090	777	9	28
pdac22	p022	756	705	663	396	826	609	488	14	23
pdac26	p026	475	261	239	76	317	415	433	9	15
pdac27	p027	628	377	334	106	438	627	470	75	29
pdac32	p032	694	461	486	209	575	463	300	35	26
pdac39	p039	509	367	319	122	528	923	624	15	15
pdac47	p047	774	399	351	162	683	602	638	116	29
pdac48	p048	574	414	420	151	654	745	459	0	18
pdac64	p064	637	446	279	65	405	596	380	0	48
pdac72	p072	1047	655	586	212	645	797	660	36	0
pdac75	p075	906	750	578	234	742	526	430	36	38
pdac81	p081	588	431	349	95	332	1159	1095	7	31
pdac83	p083	662	351	449	194	564	1065	609	1	38
pdac89	p089	552	385	466	146	499	917	519	70	20
pdac100	p100	1251	639	809	213	957	966	414	51	57

Supplementary Table 6: Drug screen results

Organoid line	Drug	LD50 (nM)	AUC	AUCpi
p003	5-FU	38548.70117	0.20348353	0.48076149
p003	Erlotinib	>50000	-0.0809063	0.08393479
p003	Gemcitabine	23.50963641	0.34870862	0.44527196
p003	Irinotecan	392.9005332	0.20790709	0.33156701
p003	Oxaliplatin	9328.213107	0.04123505	0.12885448
p003	Paclitaxel	16.88867821	0.10898313	0.1619454
p018	5-FU	448.7449095	0.22683951	0.32393168
p018	Erlotinib	>50000	-0.0386579	-0.005846
p018	Gemcitabine	71.31801182	0.11525907	0.33244749
p018	Irinotecan	>10000	0.02086252	0.28995562
p018	Oxaliplatin	>100000	0.01695898	0.09993713
p018	Paclitaxel	0.974763298	0.16314415	0.14650774
p019	5-FU	>1000000	0.18944375	0.38341918
p019	Erlotinib	>50000	0.07124501	0.10980103
p019	Gemcitabine	6.6023902	0.26534173	0.60793596
p019	Irinotecan	1677.586744	0.05361906	0.22168002
p019	Oxaliplatin	>100000	0.03155313	0.12951181
p019	Paclitaxel	2.814887155	0.2329745	0.43378279
p022	5-FU	>1000000	0.09902425	0.27659361
p022	Erlotinib	>50000	-0.2177626	-0.070515
p022	Gemcitabine	4.801560958	0.32198008	0.39532269
p022	Irinotecan	3456.790846	0.06483179	0.1198497
p022	Oxaliplatin	>100000	0.06674479	0.19057898
p022	Paclitaxel	>500	0.01332512	0.04394495
p026	5-FU	440856.9126	0.10254431	0.41958057
p026	Erlotinib	>50000	-0.0413778	0.06837519
p026	Gemcitabine	23.55937136	0.15190239	0.57408838
p026	Irinotecan	700.7689271	0.03385803	0.02623816
p026	Oxaliplatin	>100000	0.03365012	0.24723966
p026	Paclitaxel	3.046008316	0.18879533	0.26161886
p027	5-FU	219635.2373	0.18389908	0.47454342
p027	Erlotinib	>50000	0.01848314	0.13833455
p027	Gemcitabine	16.65151833	0.28668723	0.52291025
p027	Irinotecan	2377.290411	0.06848224	0.17113535
p027	Oxaliplatin	>100000	0.04875948	0.20429066
p027	Paclitaxel	>500	0.17154402	0.3473504
p029	5-FU	>1000000	0.10909178	0.23622962
p029	Erlotinib	>50000	-0.20924	0.00337301

p029	Gemcitabine	>5000	0.08732938	0.20248099
p029	Irinotecan	>10000	0.01966243	0.25056006
p029	Oxaliplatin	>100000	0.03879516	0.33134523
p029	Paclitaxel	>500	0.07231921	0.15248895
p032	5-FU	>1000000	0.1573822	0.3742463
p032	Erlotinib	>50000	0.05767189	-0.0111187
p032	Gemcitabine	13.65404284	0.22326615	0.3398206
p032	Irinotecan	1821.885666	0.12220841	0.13570944
p032	Oxaliplatin	>100000	0.02874106	0.05952196
p032	Paclitaxel	5.766470248	0.23689854	0.2282412
p037	5-FU	>1000000	-0.1449885	0.24641915
p037	Erlotinib	>50000	-0.2838531	0.13236281
p037	Gemcitabine	15.50914877	0.05522769	0.53199888
p037	Irinotecan	>10000	0.00335313	0.35460898
p037	Oxaliplatin	>100000	-0.0625552	0.01580286
p037	Paclitaxel	>500	-0.2934191	0.18195803
p039	5-FU	>1000000	-0.0291775	0.17002328
p039	Erlotinib	>50000	-0.2921333	-0.0334412
p039	Gemcitabine	>5000	0.09240161	0.5433918
p039	Irinotecan	>10000	0.00728908	0.34198607
p039	Oxaliplatin	>100000	0.00766854	0.13270278
p039	Paclitaxel	>500	0.01714926	0.13193397
p042	5-FU	>1000000	0.03706548	-0.0369554
p042	Erlotinib	>50000	-0.0332965	-0.0949649
p042	Gemcitabine	1.497948514	0.12827077	0.08081894
p042	Irinotecan	>10000	-0.0765865	-0.0441032
p042	Oxaliplatin	>100000	0.01596835	-0.0341358
p042	Paclitaxel	0.994572601	0.10940922	-0.0582745
p047	5-FU	>1000000	0.10940477	0.56410072
p047	Erlotinib	>50000	-0.0759938	-0.014399
p047	Gemcitabine	71.8693769	0.12961415	0.49327084
p047	Irinotecan	>10000	0.03921367	0.37015569
p047	Oxaliplatin	>100000	-0.0965368	-0.0065654
p047	Paclitaxel	3.753482456	0.1071899	0.28491396
p048	5-FU	>1000000	0.29688209	0.40106359
p048	Erlotinib	>50000	0.01939404	0.03008693
p048	Gemcitabine	7.803481663	0.4212718	0.5912397
p048	Irinotecan	1370.097964	0.15022347	0.29547151
p048	Oxaliplatin	32727.04017	0.08888248	0.26922748
p048	Paclitaxel	>500	0.10210168	0.13457331
p064	5-FU	>1000000	0.01972833	0.27607503
p064	Erlotinib	>50000	-0.1676786	-0.0107646

p064	Gemcitabine	21.38953294	0.08415512	0.49354293
p064	Irinotecan	>10000	0.00130197	0.17945634
p064	Oxaliplatin	>100000	-0.0100034	0.15258719
p064	Paclitaxel	>500	0.03010797	0.19606931
p072	5-FU	>1000000	-0.0322539	0.37550355
p072	Erlotinib	>50000	-0.1019458	0.05607649
p072	Gemcitabine	>5000	0.11113167	0.60792136
p072	Irinotecan	>10000	-0.0050741	0.31729172
p072	Oxaliplatin	>100000	-0.0407986	0.2654126
p072	Paclitaxel	>500	-0.0079819	0.45374251
p073	5-FU	575234.2675	0.10164677	0.3401449
p073	Erlotinib	>50000	-0.0348204	-0.0437875
p073	Gemcitabine	27.15242171	0.17610188	0.39588647
p073	Irinotecan	>10000	0.03093332	0.21776111
p073	Oxaliplatin	>100000	0.00476662	0.1651609
p073	Paclitaxel	81.27059946	0.08529981	0.23918647
p075	5-FU	>1000000	0.05970846	0.21473405
p075	Erlotinib	>50000	0.05590189	-0.0240642
p075	Gemcitabine	>5000	0.2078726	0.28138315
p075	Irinotecan	>10000	0.02647244	0.04487764
p075	Oxaliplatin	>100000	0.0436095	-0.0276198
p075	Paclitaxel	1.892819871	0.0835769	0.12830437
p089	5-FU	251531.3997	0.08558049	0.49399363
p089	Erlotinib	922.0088513	0.13006466	-0.0137774
p089	Gemcitabine	35.81715989	0.22061377	0.46113207
p089	Irinotecan	977.5070683	0.15028888	0.20595782
p089	Oxaliplatin	>100000	-0.0249884	0.27850293
p089	Paclitaxel	>500	0.11597065	0.32613667
p080	5-FU	>1000000	0.09077908	0.51859739
p080	Erlotinib	>50000	-0.0783702	0.07188214
p080	Gemcitabine	20.8649001	0.24951906	0.61277687
p080	Irinotecan	>10000	0.03039478	0.36440437
p080	Oxaliplatin	>100000	0.003941	0.25306581
p080	Paclitaxel	5.510331545	0.10973534	0.32181942
p081	5-FU	>1000000	0.07055395	0.53298704
p081	Erlotinib	>50000	0.01549338	-0.0058138
p081	Gemcitabine	14.97655969	0.14573419	0.63626715
p081	Irinotecan	>10000	0.0209675	0.15320225
p081	Oxaliplatin	>100000	0.02762664	0.25397809
p081	Paclitaxel	0.284162454	0.11372972	0.27265335
p083	5-FU	>1000000	0.02014913	0.35605688
p083	Erlotinib	>50000	-0.0973583	0.10536792

p083	Gemcitabine	>5000	0.13054216	0.35158251
p083	Irinotecan	105.8585452	0.06344216	0.27389683
p083	Oxaliplatin	>100000	-0.1544317	0.09833047
p083	Paclitaxel	13.12520573	0.12646858	0.18154911
p084	5-FU	>1000000	0.06851155	0.4189277
p084	Erlotinib	>50000	-0.0763448	0.17680915
p084	Gemcitabine	12.98464136	0.18186443	0.74926666
p084	Irinotecan	>10000	-0.0343899	0.23895617
p084	Oxaliplatin	>100000	0.00291628	0.43863125
p084	Paclitaxel	>500	-0.0496647	0.24797173
p085	5-FU	>1000000	-0.0346051	0.27243095
p085	Erlotinib	>50000	-0.0557604	0.12157033
p085	Gemcitabine	>5000	0.17201665	0.49947793
p085	Irinotecan	>10000	0.02570457	0.18306737
p085	Oxaliplatin	>100000	0.05034892	0.08555182
p085	Paclitaxel	>500	-0.0197087	0.1649811
p100	5-FU	>1000000	0.04424599	0.60997112
p100	Erlotinib	>50000	-0.1750589	-0.070946
p100	Gemcitabine	>5000	0.07487828	0.31135496
p100	Irinotecan	>10000	0.04757756	0.16417696
p100	Oxaliplatin	>100000	-0.029211	0.19470804
p100	Paclitaxel	4.24225602	0.08465656	0.16116942

Supplementary References:

1. Zhang, M. *et al.* Characterising cis-regulatory variation in the transcriptome of histologically normal and tumour-derived pancreatic tissues. *Gut* **67**, 521–533 (2018).
2. Moffitt, R. A. *et al.* Virtual microdissection identifies distinct tumor- and stroma-specific subtypes of pancreatic ductal adenocarcinoma. *Nat. Genet.* **47**, 1168–1178 (2015).
3. Collisson, E. A., Bailey, P., Chang, D. K. & Biankin, A. V. Molecular subtypes of pancreatic cancer. *Nat. Rev. Gastroenterol. Hepatol.* **16**, 207–220 (2019).
4. Szklarczyk, D. *et al.* STRING v11: Protein-protein association networks with increased coverage, supporting functional discovery in genome-wide experimental datasets. *Nucleic Acids Res.* **47**, D607–D613 (2019).
5. Saelens, W., Cannoodt, R., Todorov, H. & Saeys, Y. A comparison of single-cell trajectory inference methods. *Nat. Biotechnol.* **37**, 547–554 (2019).
6. Peng, J. *et al.* Single-cell RNA-seq highlights intra-tumoral heterogeneity and malignant progression in pancreatic ductal adenocarcinoma. *Cell Res.* (2019) doi:10.1038/s41422-019-0195-y.
