# Supplementary material for: Single-cell analysis of patient-derived PDAC organoids reveals cell state heterogeneity and a conserved developmental hierarchy
Source: Nat Commun. 2021 Oct 5;12:5826. doi: 10.1038/s41467-021-26059-4 (PMC8492851; doi:10.1038/s41467-021-26059-4)
Supplement: Supplementary file 2 — Reporting Summary [file 41467_2021_26059_MOESM2_ESM.pdf]

nature research

Corresponding author(s): Christian Conrad
Roland Eils
Oliver Strobel

Last updated by author(s): May 21, 2021

Reporting Summary

Nature Research wishes to improve the reproducibility of the work that we publish. This form provides structure for consistency and transparency in reporting. For further information on Nature Research policies, see our Editorial Policies and the Editorial Policy Checklist.

Statistics

For all statistical analyses, confirm that the following items are present in the figure legend, table legend, main text, or Methods section.

n/a	Confirmed	
☐	☒	The exact sample size (*n*) for each experimental group/condition, given as a discrete number and unit of measurement
☐	☒	A statement on whether measurements were taken from distinct samples or whether the same sample was measured repeatedly
☐	☒	The statistical test(s) used AND whether they are one- or two-sided<br>*Only common tests should be described solely by name; describe more complex techniques in the Methods section.*
☐	☒	A description of all covariates tested
☐	☒	A description of any assumptions or corrections, such as tests of normality and adjustment for multiple comparisons
☐	☒	A full description of the statistical parameters including central tendency (e.g. means) or other basic estimates (e.g. regression coefficient) AND variation (e.g. standard deviation) or associated estimates of uncertainty (e.g. confidence intervals)
☐	☒	For null hypothesis testing, the test statistic (e.g. *F*, *t*, *r*) with confidence intervals, effect sizes, degrees of freedom and *P* value noted<br>*Give P values as exact values whenever suitable.*
☒	☐	For Bayesian analysis, information on the choice of priors and Markov chain Monte Carlo settings
☒	☐	For hierarchical and complex designs, identification of the appropriate level for tests and full reporting of outcomes
☐	☒	Estimates of effect sizes (e.g. Cohen's *d*, Pearson's *r*), indicating how they were calculated

Our web collection on statistics for biologists contains articles on many of the points above.

Software and code

Policy information about availability of computer code

Data collection	Drug screen data was acquired using the "DeathPro" KNIME workflow, as published in Jabs et al. Mol Syst Biol 2019. No further third-party software was used for data acquisition.
Data analysis	Data were analyzed using CellRanger v2.1.1 (10x Genomics) as well as Seurat v3.0 in R v3.6.3, together with the uwot package v0.1.5 in R, umap-learn v0.3.10, velocyto v0.17.16 and dynverse v0.1.1, as described in detail in the Methods section.

For manuscripts utilizing custom algorithms or software that are central to the research but not yet described in published literature, software must be made available to editors and reviewers. We strongly encourage code deposition in a community repository (e.g. GitHub). See the Nature Research guidelines for submitting code & software for further information.

Data

Policy information about availability of data

All manuscripts must include a data availability statement. This statement should provide the following information, where applicable:

- Accession codes, unique identifiers, or web links for publicly available datasets
- A list of figures that have associated raw data
- A description of any restrictions on data availability

All scRNA-seq data generated in this study has been uploaded to the EGA portal (accession number EGAS00001004661). Primary PDAC scRNA-seq data was downloaded from the GSA (accession number CRA001160, samples T18 and T20). Gene ontology (GO) term analysis was performed using the MSigDB database v7.1 (https://www.gsea-msigdb.org). Gene interactions networks were identified using the STRING database v11 (https://string-db.org).

April 2020

Field-specific reporting

Please select the one below that is the best fit for your research. If you are not sure, read the appropriate sections before making your selection.

- [x] Life sciences
- [ ] Behavioural & social sciences
- [ ] Ecological, evolutionary & environmental sciences

For a reference copy of the document with all sections, see nature.com/documents/nr-reporting-summary-flat.pdf

Life sciences study design

All studies must disclose on these points even when the disclosure is negative.

Sample size	Sample size was based on availability of patient biopsies (number of patients who presented with PDAC for surgery and consented to samples being included in an organoid biobank). This sample size provided a sufficiently large number of cells for the purpose of exploring cellular heterogeneity across patients (> 90 000 cells, comparable to other current studies of similar design).
Data exclusions	No data were excluded from the analysis.
Replication	We aimed to ensure reproducibility by including patient-derived organoids from a large set of patients in this study. Single-cell RNA sequencing experiments were performed once for each sample. FISH stainings were repeated twice on individual tissue sections and results were replicated successfully.
Randomization	No randomization into experimental groups was required for this study because samples were not subjected to differential treatments.
Blinding	Blinding of investigators was not necessary for this study because all samples were analysed in the same way and were not subjected to differential treatments.

Reporting for specific materials, systems and methods

We require information from authors about some types of materials, experimental systems and methods used in many studies. Here, indicate whether each material, system or method listed is relevant to your study. If you are not sure if a list item applies to your research, read the appropriate section before selecting a response.

Materials & experimental systems

n/a	Involved in the study	
x		Antibodies
x		Eukaryotic cell lines
x		Palaeontology and archaeology
x		Animals and other organisms
	x	Human research participants
x		Clinical data
x		Dual use research of concern

Methods

n/a	Involved in the study	
x		ChIP-seq
x		Flow cytometry
x		MRI-based neuroimaging

Human research participants

Policy information about studies involving human research participants

Population characteristics	Samples were derived from 8 male and 12 female patients. Information on patient age was not available for this study. All patients carried a KRAS mutation. Two patients had received chemotherapy prior to surgery, although no further information on the therapy regimen was available for this study.
Recruitment	Participants were recruited based on presenting for surgery with PDAC and providing consent to samples being included in an organoid biobank. No further pre-selection of participants was performed, and no potential biases apply.
Ethics oversight	The study protocol for organoid generation was approved by the Ethics Committee of Heidelberg University (ethic votes 301/2001, 159/2002, S-206/2011, S-708/2019).

Note that full information on the approval of the study protocol must also be provided in the manuscript.
